# Why were some countries more successful than others in curbing early COVID-19 mortality impact? A cross-country configurational analysis

**DOI:** 10.1371/journal.pone.0282617

**Published:** 2023-03-08

**Authors:** Bin Chen, Yao Liu, Bo Yan, Long Wu, Xiaomin Zhang

**Affiliations:** 1 Marxe School of Public and International Affairs, Baruch College & The Graduate Center, The City University of New York, New York, New York, United States of America; 2 School of Public Policy and Administration, Xi’an Jiaotong University, Xi’an, Shaanxi, China; Nanyang Technological University, SINGAPORE

## Abstract

Why was there considerable variation in initial COVID-19 mortality impact across countries? Through a configurational lens, this paper examines which configurations of five conditions—a delayed public-health response, past epidemic experience, proportion of elderly in population, population density, and national income per capita—influence early COVID-19 mortality impact measured by years of life lost (YLL). A fuzzy-set qualitative comparative analysis (fsQCA) of 80 countries identifies four distinctive pathways associated with high YLL rate and four other different pathways leading to low YLL rate. Results suggest that there is no singular “playbook”—a set of policies that countries can follow. Some countries failed differently, whereas others succeeded differently. Countries should take into account their situational contexts to adopt a holistic response strategy to combat any future public-health crisis. Regardless of the country’s past epidemic experience and national income levels, a speedy public-health response always works well. For high-income countries with high population density or past epidemic experience, they need to take extra care to protect elderly populations who may otherwise overstretch healthcare capacity.

## 1. Introduction

As of January 1, 2023, the COVID-19 pandemic had already infected 733,303,027 people, causing 6,697,563 deaths globally [1]. Either fatality or mortality has been used to measure the COVID-19 death rate around the world. Fatality is the proportion of people who die among all diagnosed with COVID-19. With underreported infected individuals in some countries due to their limited testing capacities, fatality is likely to be underestimated [2]. Mortality, isolating the effect of testing capacity, serves as a better outcome measure when assessing the effects of countries’ early COVID-19 responses [3,4]. However, mortality, computed based on reported deaths, cannot provide sufficient information on how many years or weeks of life have been lost due to the disease [5]. Years of life lost (YLL) and weeks of life lost (WLL) are two preferable indicators to assess the mortality impact of COVID-19 [6–9].

A large number of studies employed the YLL method to estimate the mortality impact of COVID-19 [5–7], while the WLL method, a relatively new approach, was only used to measure the COVID-19 mortality burden in France, the United Kingdom, and the United States [8,9]. Because of the availability of data, we used early YLL rate in this study, which was measured by the number of years of life lost due to COVID-19 per 100,000 people as of January 6, 2021 [5]. What is striking is that early YLL rates varied sharply from country to country. On the one hand, the YLL rate was under 10 in Ethiopia, Cameroon, China, Burkina Faso, New Zealand, Bolivia, Iraq, Japan, and Chad; and on the other hand, above 1,000 in Peru, Mexico, Colombia, Brazil, Belgium, the United States, Chile, Slovenia, Ecuador, and the United Kingdom.

Why were some countries more successful than others in curbing YLL rate in the early stage of the virus? This question is a puzzle that has spawned numerous theories and speculations but no definitive answers. Studies underway around the world are looking into how 1) a delayed public-health response, 2) past epidemic experience, 3) elderly populations, 4) population density, and 5) national income per capita might have affected a wide variation in early mortality impact. Each possible explanation comes with confounding counterevidence. Different from medical research, our study focuses on these five contributors concerning public-health response policy, demographic, and economic factors.

### 1.1 Pandemic response policy

#### A delayed public-health response

Policy response is a critical factor in explaining the differences in epidemic control across countries [10,11]. Early and quick public-health responses, such as social-distancing and lockdown measures, have clearly been effective [12,13]. It is believed that a delayed public-health response leads to more deaths [14,15]. Countries that locked down early, such as Singapore and New Zealand, have been able to avoid out-of-control contagions [16,17]. Counterintuitively, some countries where the authorities reacted late and the enforcement of lockdowns was uneven appear to have been spared: Cameroon and Bolivia, for instance, had low YLL rates—0.85 and 7.04 per 100,000 population respectively—even though both of these countries reacted late. An ongoing debate is whether countries should respond quickly without sufficient information or to wait until related knowledge about the virus is available [18].

#### Past epidemic experience

Experience of handling prior epidemics, no matter success or failure, may improve countries’ policy learning capability in response to the next crisis [19]. Asian countries, such as South Korea, successfully managed the outbreak of coronavirus because they had been affected by earlier epidemics of SARS and MERS [20]. From that experience, they learned lessons, and thus they were quick to adopt different measures, such as widespread testing, proactive tracing of infected people, and treatment of infected people, in response to the COVID-19 outbreak [20]. In Africa, countries with bitter experience of HIV, drug-resistant tuberculosis, and Ebola also reacted quickly [21]. However, countries with more prior epidemic experience are also more vulnerable to subsequent outbreaks. For instance, the United States, which had previous experience with many infectious diseases, such as SARS and MERS, performed poorly in curbing the early YLL rate [6].

### 1.2 Demographics

#### Proportion of elderly in population

Demographic factors are regarded as decisive conditions influencing the transmission and control of COVID-19 [22]. Evidence shows that COVID-19 is highly lethal in the elderly (age above 65), especially those with underlying medical conditions [23]. Many countries have escaped a high number of COVID-19 deaths due to their relatively younger populations [24]. Africa—with 42,750 reported COVID-19 deaths as of January 1, 2021, a tiny fraction of its 1.3 billion population—is the world’s youngest continent, with 60 percent of its population being under the age of 25 [25]. By contrast, the national median age in Italy, one of the hardest hit countries, is more than 45 [25]. The mean age of those who died of COVID-19 in Italy was 81 years [26], and most of these patients had at least one comorbidity before being infected [27]. Yet there are notable exceptions to this demographic theory. Japan, with the world’s oldest population, has had a lower number of casualties due to its successful protection of the elderly [28]. Ecuador has been the worst hit country in the Latin American region [29], although it is one of the youngest countries in the world, with only 7.4 percent of its residents aged over 65 years [25].

#### Population density

Population density has been seen as a facilitator of contagion and social pathologies of many forms [30]. The sheer density of the population will be easier suffering from COVID-19 outbreak, because the risks of infections eruption from epicenters may emerge due to higher population mobility and connectivity [31]. Cross-country studies have shown that higher population density would increase the likelihood of inter-community contagion, thus causing more deaths [32,33]. However, having more people in less space does not necessarily equate risk [34]. For instance, Singapore, one of the most densely populated countries in the world, has contained the pandemic well due to its early quick response and health system resilience [35]. In contrast, Peru, with a very low population density (25.4 people per square kilometer), achieved a high YLL rate of 2,319.73. Therefore, population density plays a vital role in the transmission of COVID-19, but this role should be scrutinized under specific socioeconomic contexts [36].

### 1.3 National wealth

#### National income per capita

National wealth gap, usually measured by national income per capita, is another determinant that should be taken into account when analyzing the COVID-19 abatement among different countries [36]. It was believed that high-income countries can cope better with the pandemic because of their higher investment in medical infrastructure and financial relief [36]. However, research has demonstrated a positive relationship between economic resources and YLL in the early wave of COVID-19 [37]. For instance, while high-income countries such as the United States, Belgium, and the United Kingdom have much higher national income per capita than low-income countries including Burkina Faso, Chad, and Sierra Leone, the former witnessed one hundred times higher YLL rate than the latter [5]. The debatable relationship between economic disparities and early COVID-19 mortality impact still needs to be explored with the combination of policy and demographic factors.

These inconsistencies are largely due to the fact that there may be no single reason for some countries being hit and others being missed. The answer is likely to be some combinations of the above factors. Most studies, in particular the ones using quantitative methods, consider different factors to have independent effects on the COVID-19 mortality impact, overlooking the interaction of multiple factors [38,39]. Moreover, these factors may combine in specific ways when inducing mortality impact. This study addresses the following research question: Under what configurations of these five factors have countries encountered high or low YLL rates?

This paper aims to shed some light on these combined effects by applying a configurational approach using a fuzzy-set qualitative comparative analysis (fsQCA) method. It has been almost three years since the World Health Organization declared the COVID-19 outbreak a global pandemic—a crisis that has profoundly affected lives and livelihoods. By reflecting on the many challenges and lessons of the past years, organizations, governments, and societies can continue the journey toward normality and emerge stronger on the other side.

## 2. Methods

The fsQCA method is based on the principles of set theory, formal logic, and Boolean and fuzzy algebra. It uses a specific terminology: *condition* is used instead of independent variable, *outcome* instead of dependent variable, and *cases* instead of observations, and results are called *solutions*, *pathways*, *configurations*, or *recipes* [40]. An fsQCA allows us to study which set of conditions is necessary and sufficient to bring about a certain outcome [41]. The assessment of causal complexity in set-theoretic methods is based on three assumptions: 1) conjunctural causation—a condition only has an effect in combination with other conditions; 2) equifinality—multiple, mutually nonexclusive paths lead to the same outcome; and 3) causal asymmetry—the presence of an outcome may have other explanations than its absence (e.g., high versus low YLL rate) [40]. The fsQCA method is also highly appropriate for analyzing small-to-medium N cases [42].

The interpretation of the results is mainly based on the *consistency* and *coverage* values indicated in the solutions. Consistency shows the extent to which the involved solution path is consistent with reality, or, in other words, the extent to which this solution path leads to the outcome. Coverage, by contrast, assesses the degree to which a cause or causal combination accounts for an outcome. Hence, coverage reports the proportion of membership to the outcome explained by the overall solution term, indicating the percentage of the cases covered. The threshold for the consistency ratio is set to no less than 0.75 [41]. In this study, we set the higher consistency threshold at 0.80 and the frequency cutoff at 1 [38,43]. While fsQCA software generates three types of solution formulae (complex, parsimonious, and intermediate), we apply the intermediate solution, which is in-between the complex and most parsimonious solutions in terms of complexity [38]. The intermediate solution is suggested as the main point of reference for interpreting QCA results [41].

### 2.1 Data collection and measurement

With data gleaned from the Oxford COVID-19 Government Response Tracker (OxCGRT), the World Health Organization, the World Bank, and the study of Pifarré i Arolas et al., we constructed a dataset of 80 countries on the basis of availability of data for a cross-country comparative configurational analysis. Our study comprises *YLL rate* as the outcome of interest and five explanatory conditions: *a delayed public-health response*, *past epidemic experience*, *proportion of elderly in population*, *population density*, and *national income per capita*. Table 1 presents the descriptive statistics of the five conditions and the outcome, and the dataset of 80 countries is provided in S1 Table.

**Table 1:** Descriptive statistics of conditions and outcome.

Conditions and Outcome	Mean	Standard Deviation	Minimum	Maximum
**Conditions**				
A delayed public-health response	50.36	21.02	0	79
Past epidemic experience	0.58	0.50	0	1
Proportion of elderly in population	12.53	6.85	2.41	28
Population density	164.41	238.22	3.30	1,575.19
National income per capita	21,073	22,217.83	440	83,280
**Outcome**				
YLL rate	412.04	539.92	0.07	2,319.73

*YLL rate* was computed as the number of years of life lost due to COVID-19 per 100,000 population as of January 6, 2021, obtained directly from the study of Pifarré i Arolas et al. [5]. With a focus on early COVID-19 mortality impact, this study used January 6, 2021 as the cutoff point to isolate the effects of vaccination rollout [44], the availability of more effective treatments, and the emergence of new virus variants on overall COVID-19 mortality impact.

*A delayed public-health response* concerned the number of days from January 1, 2020 until a country first implemented any of nine containment and closure policies [45]. Specifically, OxCGRT provides researchers with nine indicators recording information on the enactment of containment and closure policies: school closing, workplace closing, cancel public events, restrictions on gatherings, close public transport, stay at home requirements, restrictions on internal movement, international travel controls, and facial coverings [46].

*Past epidemic experience* was a binary condition (0 or 1) showing whether a country had experienced one of four recent epidemics: SARS, MERS, Zika, and Ebola [47]. The epidemiological records on the above four pandemics were all collected from the World Health Organization.

*Proportion of elderly in population* was computed as the population aged 65 years and above as a percentage of the total population in a country [48]. *Population density* was midyear population divided by land area in square kilometers [49]. *National income per capita* referred to the gross national income, converted to U.S. dollars using the World Bank Atlas method, divided by the midyear population [50]. All of these three conditions were obtained directly from the World Bank.

### 2.2 Calibration

In QCA, cases receive membership scores, ranging from 0 to1, for each condition and outcome, which are all sets. These membership scores are calibrated to reflect the presence of a case in a certain set of conditions or outcomes. Calibration can be either direct or indirect, the latter needing a stronger theoretical basis than the former [38]. Considering the lack of theory and substantive knowledge guiding the key thresholds on the outcome and conditions, this study employed a direct fuzzy-set method to allow conditions and the outcome to display different degrees of membership in sets. The membership scores of cases in each set were generated in the calibration procedure based on the distribution of raw case values [40]. Three anchor points defined a set: full membership (the 95^th^ percentile), full nonmembership (the 5^th^ percentile), and a crossover point (the 50^th^ percentile) [38]. Then, the raw data were transformed into the log-odds metric, with all values ranging from 0 to 1.

Taking early YLL rate as an example, the thresholds of full membership (the 95^th^ percentile), crossover point (the 50^th^ percentile), and full nonmembership (the 5^th^ percentile) were set at 1,428.78, 166.52, and 6.35, respectively. Any raw value above 166.52 was assigned a membership score between 1 and 0.5, whereas any raw value below 166.52 was calibrated to a membership score between 0.5 and 0. Argentina scored 1,172.82 on raw YLL rate, and its fuzzy-set membership score was calibrated as 0.921. Algeria scored 35.14 on raw YLL rate, and its fuzzy-set membership score was calibrated as 0.081. The calibration thresholds are provided in Table 2, and the fuzzy-set membership scores of the 80 countries are provided in S2 Table.

**Table 2:** Calibration thresholds.

	Full Membership (95^th^ Percentile)	Crossover Point (50^th^ Percentile)	Full Nonmembership (5^th^ Percentile)
A delayed public-health response	75	56.50	20.90
Past epidemic experience	1	/	0
Proportion of elderly in population	21.95	13.13	2.64
Population density	462.36	99.06	10.31
National income per capita	63,703.50	12,200	746.50
YLL rate	1,428.78	166.52	6.35

### 2.3 Data analysis

We first performed an analysis of necessity to test whether any single condition and its negation was necessary for either a high or low YLL rate. The results in S3 Table show that none of the five conditions (neither in its absence nor in its presence) reached the minimum consistency benchmark of 0.9 for necessity [51], suggesting that no single condition alone can explain high or low YLL rates. This underscores the importance of exploring the combinations of conditions resulting in high and low YLL rates.

We now turn to the analysis of combinations of the five conditions sufficient for high and low YLL rates. We ran the fuzzy-set algorithm and obtained the truth tables. The truth tables displaying the configurations associated with high and low YLL rates are presented in S4 and S5 Tables. In the truth tables, we find no contradictory or deviant cases. We performed the standard analyses to generate the complex, parsimonious, and intermediate solutions.

In producing the intermediate solutions, we had to decide how each causal condition should theoretically and empirically contribute to the outcome. Specifically, based on the existing research findings, we expected that the presence of *a delayed public-health response* and *a large proportion of elderly in the population* would contribute to a high YLL rate, whereas the absence of *a delayed public-health response* and *a small proportion of elderly in the population* would contribute to a low YLL rate. When it comes to *past epidemic experience*, one would expect that it will lead to a low YLL rate. However, regression analysis suggests the opposite direction (regression results are provided in S6 Table). Therefore, we expected that the presence of *past epidemic experience* would contribute to a high YLL rate, whereas the absence of *past epidemic experience* would contribute to a low YLL rate.

We further discerned the core and peripheral conditions by examining the parsimonious and intermediate solutions together. Core elements indicate a strong causal relationship with the outcome, and peripheral elements indicate a weaker relationship [52]. The conditions that appear in both parsimonious and intermediate solutions are core conditions, whereas conditions that are not included in the parsimonious solutions and only appear in the intermediate solutions are peripheral conditions [52].

## 3. Results

Tables 3 and 4 show the configurations for high and low YLL rates, respectively, where a black circle ● indicates the presence of a core condition; a crossed-out circle ⊗ indicates the absence of a core condition; a black square ■ indicates the presence of a peripheral condition; a crossed-out square ⌧ indicates the absence of a peripheral condition; and a blank space indicates a “don’t care” condition [52]. The “don’t care” situation indicates that the condition does not play a role in a specific configuration.

**Table 3:** Configurations for high YLL rate.

Conditions	High YLL rate			
	1	2	3	4
A delayed public-health response				■
Past epidemic experience		⊗	■	■
Proportion of elderly in population		●	⊗	
Population density	●	●		
National income per capita	●		●	■
Consistency	0.79	0.76	0.84	0.90
Raw Coverage	0.49	0.23	0.21	0.30
Unique Coverage	0.05	0.03	0.02	0.04
Countries	Belgium; United Kingdom; **Switzerland**; **Italy**; Czech Republic; **France**; Portugal; Slovak Republic; **Austria**	Belgium; Czech Republic; Portugal; Slovak Republic; **Albania**; **Slovenia**	Chile; **Panama**; Argentina	United Kingdom; **Spain**; **Sweden**; **Greece**; Chile; Argentina
Overall Solution Consistency	0.79			
Overall Solution Coverage	0.63			

Note: ● = core condition present; ⊗ = core condition absent; ■ = peripheral condition present; ⌧ = peripheral condition absent; blank space = a “don’t care” condition.

**Table 4:** Configurations for low YLL rate.

Conditions	Low YLL rate			
	5	6	7	8
A delayed public-health response			⊗	⌧
Past epidemic experience	⊗		⊗	●
Proportion of elderly in population		⊗		●
Population density	⊗	●		
National income per capita		⌧	●	⊗
Consistency	0.81	0.86	0.74	0.85
Raw Coverage	0.33	0.40	0.14	0.13
Unique Coverage	0.15	0.18	0.03	0.04
Countries	**Latvia**; **Afghanistan**; **Iraq**; **Kenya**	**Pakistan**; **El Salvador**; **Nigeria**; **Nepal**; **Malawi**; **China**; **Togo**; **Sierra Leone**; **Ethiopia**	**Japan**; **Croatia**	**Cuba**; **Romania**
Overall Solution Consistency	0.80			
Overall Solution Coverage	0.67			

Note: ● = core condition present; ⊗ = core condition absent; ■ = peripheral condition present; ⌧ = peripheral condition absent; blank space = a “don’t care” condition.

### 3.1 Configurations for high YLL rate

Table 3 shows four configurations of conditions that are sufficient to explain a high YLL rate, representing an overall solution consistency ratio (0.79) and an overall solution coverage ratio (0.63) that accounts for a substantial proportion of the outcome. The fsQCA also estimates the empirical relevance of every solution by calculating raw and unique coverage. The raw coverage describes the amount of the outcome that is explained by a certain alternative solution, while the unique coverage describes the amount of the outcome that is exclusively explained by a certain alternative solution. The overlap across the four pathways is high, as indicated by the low unique coverage. It suggests that the four configurations are not mutually exclusive, an important feature of QCA. Many countries, for example, the United Kingdom, can fit pathways 1 and 4, whereas a few other countries (highlighted in bold in Table 3) are uniquely covered by a particular pathway (e.g., Switzerland in Configuration 1).

Configuration 1 covers nine countries, representing a combination of high population density and high national income per capita. In Configuration 2, six countries have the combination of the absence of past epidemic experience, a large proportion of elderly in the population, and high population density. In Configuration 3, three countries align with the combination of the presence of past epidemic experience, a small proportion of elderly in the population, and high national income per capita. Configuration 4 covers six countries, representing a combination of three peripheral conditions including a delayed public-health response, the presence of past epidemic experience, and high national income per capita.

### 3.2 Configurations for low YLL rate

The analysis of low YLL rate (Table 4) generated four configurations of conditions sufficient for a low YLL rate. In comparison to the analysis of high YLL rate, it yielded similar overall solution consistency (0.80) and coverage ratio (0.67).

Configuration 5 illustrates a combination of the absence or low level of two core conditions: the absence of the past epidemic and low population density, covering four countries. In Configuration 6, nine countries fall into the combination of the inverse of one peripheral condition (low national income per capita), the opposite of one core condition (a small proportion of elderly in the population), and the presence of one core condition (high population density). Two countries share Configuration 7 composed of three core conditions (a swift public-health response, no past epidemic experience, and high national income per capita). Configuration 8 covering two countries represents a combination of four core or peripheral conditions: a quick public-health response, past epidemic experience, a large proportion of elderly in the population, and low national income per capita.

### 3.3 Predictive validity testing and robustness analysis

Predictive validity is important because the models with a good fit do not necessarily provide accurate forecasts [53]. Hence, we tested the configurations for predictive validity as a robustness check of the above findings. To assess the predictive validity, we followed the procedure proposed by Pappas & Woodside [54]. First, we randomly divided the total cases into a subsample and a holdout sample. Next, we conducted the same analysis for the subsample as we did in the previous process. After obtaining the results from the subsample (see Table 5), we used the holdout sample to test the predictive validity of each configuration generated from the subsample [54].

**Table 5:** Configurations of the subsample for high and low YLL rates.

Conditions	High YLL rate		Low YLL rate	
	M1	M2	M3	M4
A delayed public-health response	●	●		⌧
Past epidemic experience		●		
Proportion of elderly in population	●	●	⌧	
Population density	●		●	●
National income per capita	■	■	⊗	⊗
Consistency	0.96	0.90	0.86	0.83
Raw Coverage	0.42	0.28	0.40	0.37
Unique Coverage	0.18	0.04	0.08	0.04
Overall Solution Consistency	0.93		0.83	
Overall Solution Coverage	0.46		0.45	

Note: ● = core condition present; ⊗ = core condition absent; ■ = peripheral condition present; ⌧ = peripheral condition absent; blank space = a “don’t care” condition.

Specifically, four configurations of the subsample for high and low YLL rates were regarded as four models, and each model was computed as a new condition in the holdout sample. Then, the new condition was used to plot against the outcome using the holdout sample. All plots are presented in S1 File. The plots provided two numbers of “consistency”: the larger number was consistency, while the other was coverage [54]. Neither of these should contradict the consistency and coverage of the corresponding configuration in the subsample [54]. For instance, S7a Fig in S1 File shows the plot of M1 (i.e., the combination of a delayed public-health response, a large proportion of elderly in population, high population density, and high national income per capita) of the subsample using data from the holdout sample. The consistency and coverage of the plot (0.80 and 0.32) resembled that of model 1 (0.96 and 0.42), indicating the good predictive validity of model 1. The other three models also had high predictive validity.

To test the robustness of our results in Tables 3 and 4, we altered the consistency threshold levels to 0.85 and 0.90 [55]. Tables 6 and 7 below show a comparison of configurations with different cut-offs for high and low YLL rates, respectively. When we used a cut-off at 0.85, only configurations 3, 6 and 7 are affected, reflecting that a small proportion of elderly in population does not play any role in predicting high YLL rate, whereas population density and national income per capita lose their importance in predicting low YLL rate. With a cut-off of 0.90, more configurations are affected. Nevertheless, all of the configurations at the 0.90 cut-off are subsets of those at 0.80, and most changes are seen in two causal conditions—population density and national income per capita—that were assumed to be neither present nor absent when we generated intermediate solution. Overall, additional robustness analyses yielded similar results.

**Table 6:** Comparison of configurations for high YLL rate at different cut-offs.

Configuration 1	Cut-off 0.80	Density	Income	
	Cut-off 0.85	✓	✓	
	Cut-off 0.90	✓	✓	
Configuration 2	Cut-off 0.80	~ Exp	Elderly	Density
	Cut-off 0.85	✓	✓	✓
	Cut-off 0.90	✓	✓	✓
Configuration 3	Cut-off 0.80	Exp	~ Elderly	Income
	Cut-off 0.85	✓	✘	✓
	Cut-off 0.90	✓	✘	✓
Configuration 4	Cut-off 0.80	Delay	Exp	Income
	Cut-off 0.85	✓	✓	✓
	Cut-off 0.90	✓	✓	✓

Note: Delay = A delayed public-health response; Exp = Past epidemic experience; Elderly = Proportion of elderly in population; Density = Population density; Income = National income per capita; ~ means the logical negation; ✓ = A condition appears in a configuration with a specific consistency cut-off; ✘ = A condition does not appear in a configuration with a specific consistency cut-off.

**Table 7:** Comparison of configurations for low YLL rate at different cut-offs.

Configuration 5	Cut-off 0.80	~ Exp	~ Density		
	Cut-off 0.85	✓	✓		
	Cut-off 0.90	✓	✓		
Configuration 6	Cut-off 0.80	~ Elderly	Density	~ Income	
	Cut-off 0.85	✓	✘	✓	
	Cut-off 0.90	✓	✓	✘	
Configuration 7	Cut-off 0.80	~ Delay	~ Exp	Income	
	Cut-off 0.85	✓	✓	✘	
	Cut-off 0.90	✓	✓	✘	
Configuration 8	Cut-off 0.80	~ Delay	Exp	Elderly	~ Income
	Cut-off 0.85	✓	✓	✓	✓
	Cut-off 0.90	✓	✘	✘	✘

Note: Delay = A delayed public-health response; Exp = Past epidemic experience; Elderly = Proportion of elderly in population; Density = Population density; Income = National income per capita; ~ means the logical negation; ✓ = A condition appears in a configuration with a specific consistency cut-off; ✘ = A condition does not appear in a configuration with a specific consistency cut-off.

## 4. Discussion

Since the outbreak of COVID-19, a great deal of research has been conducted about the factors that contribute to early COVID-19 mortality impact. Most studies have explored the net effects of the individual factors, including policy responses [10–13], demographics [22–24], and economic factors [36,37]. Without paying attention to the interplay of these factors, these studies produced mixed results. To address these inconsistencies, we conducted a cross-country comparative study to examine the contributing factors of YLL rate from a configurational perspective. The configurational findings confirm our premise that YLL rate is the outcome of five factors—*a delayed public-health response*, *past epidemic experience*, *proportion of elderly in population*, *population density*, *and national income per capita*—clustering into eight combinations and that none of these factors on its own is either necessary or sufficient to explain either high or low YLL rate.

### 4.1 Interpretation of configurations

The countries associated with high early YLL rates cluster into four scenarios. In the first scenario, high-income countries with high population density experienced high YLL rates. Nine typical cases of this scenario are Belgium, the United Kingdom, Switzerland, Italy, the Czech Republic, France, Portugal, the Slovak Republic, and Austria. All of these high-income countries are located in Europe and share similar demographic characteristics. A cross-country study concentrating on mortality in Europe during the first wave of COVID-19 confirmed that a 10-fold higher density was associated with 17.3 excess deaths per million habitants [56]. However, the aforementioned nine countries, even with rich economic resources, did not adopt effective policies to address the potential risks arising from the dense population, thus causing high YLL rates.

In the second scenario, a set of countries without past epidemic experience are faced with the high risks of transmission and deaths of COVID-19 due to their dense and aging populations. Six typical cases are Belgium, the Czech Republic, Portugal, the Slovak Republic, Albania, and Slovenia, among which the first four countries also exist in the first scenario. These six countries did not experience mass epidemics in the past yet facing the serious problems of dense and aging populations at the same time. The absence of epidemic experience makes it hard for them to deal with the COVID-19 crisis full of uncertainty, ambiguity, and unpredictability [57]. High levels of the aging population and population density further complicated their fight against COVID-19 [32], resulting in high YLL rates.

In the third scenario, a set of countries with past epidemic experience, young demographic structure, and relatively abundant economic resources also witnessed high YLL rates. Three typical cases are Chile, Panama, and Argentina. All of these Latin American countries have experienced outbreaks of Zika and have demographic and economic advantages in curbing COVID-19. However, these advantages did not translate into state capacity. Taking Chile as an example, Chile is a high-income country with a relatively small proportion of the elderly, and it has experienced pandemics in recent years. Nevertheless, Chile reached a high YLL rate of 1,291.46.

In the fourth scenario, a set of countries delayed their response, despite past epidemic experiences and high income levels. Six typical countries are the United Kingdom, Spain, Sweden, Greece, Chile, and Argentina. These countries have experienced pandemics such as SARS, MERS, Zika, and Ebola, along with adequate economic resources. However, all of them were hesitant to implement containment and closure policies in the early stage of the epidemic. Existing research has confirmed that richer countries took longer to take restriction measures [58,59]. For instance, the United Kingdom adopted the “herd immunity” strategy in the early phase, which greatly increased the risks of infection and caused a large number of deaths [60]. Similarly, Sweden preferred to adopt a “nudge” response strategy [61], which missed the optimal response time, causing a high YLL rate.

As regards the countries that saw low early YLL rates, they follow four different scenarios. The countries in the fifth category neither experienced epidemics in the past nor had high population density. Four typical cases in this path are Latvia, Afghanistan, Iraq, and Kenya. The population density of them is below 100 people per square kilometer [49], reducing the likelihood of transmission and thereby causing lower YLL rates. In the sixth scenario, a set of countries with high population density and low income level escaped high YLL rates due to their young demographic structure. Nine typical cases are Pakistan, El Salvador, Nigeria, Nepal, Malawi, China, Togo, Sierra Leone, and Ethiopia. All of these countries have a small proportion of elderly in their populations [48], which may weaken the high risks of population density.

In the seventh configuration, despite lacking past epidemic experience, high-income countries including Japan and Croatia responded quickly to curb the early COVID-19 mortality impact. The two countries have benefited from a combination of a quick response and abundant economic resources that compensates for their lack of prior epidemic experience. In the eighth scenario, past epidemic experience makes it easier for Cuba and Romania to take strict public-health control measures in spite of their aging populations and lack of economic resources. Different from the high-income countries in the seventh scenario, Cuba and Romania explored new avenues for countries with limited economic resources to respond to COVID-19, that is, a quick policy response in conjunction with learning from the past epidemic experience.

Overall, our study finds that high-income countries with dense and aging populations and delayed responses tend to have higher YLL rates than low-income countries with lower population density, younger populations, and quicker responses. This echoes the existing studies emphasizing the policy, demographic, and economic factors in explaining the early COVID-19 mortality impact [14,24,37]. This study enriches the existing research by providing a much more nuanced configurative perspective. For instance, high-income countries such as Japan and Croatia have escaped high YLL rates because of their early quick responses, while countries with younger demographic structures such as Chile, Panama, and Argentina have faced difficulties in responding to the pandemic.

The role of past epidemic experience in the configurations is more complicated than previous studies have found. Extant studies have suggested that the lesson or experience gained from previous epidemics is conducive for a country to fight against COVID-19 [19,20]. However, we find that past epidemic experience does not necessarily lead to better performance in terms of response to COVID-19. The countries in configurations 3 and 4 have experienced epidemics at least once before, but they all performed poorly in reducing early YLL rates. In contrast, the absence of prior epidemic experience working together with other factors contributed to low YLL rates in configurations 5 and 7. Therefore, the role of prior epidemic experience needs to be examined in conjunction with other conditions.

### 4.2 Policy implications

We highlight three policy implications derived from our configurational findings. First, a trustworthy and robust government is necessary to fight against COVID-19 [62], and governments should be held accountable for the outcome of COVID-19 [63]. To this end, governments should improve healthcare capacity, coordination capacity, and learning capacity derived from their epidemic experience. A comprehensive and holistic strategy is necessary for each country facing a pandemic like COVID-19. As is known to us all, policy capacity, social and economic profile, and demographic structure vary greatly from country to country. Thus, there is no one-size-fits-all strategy for tackling COVID-19 [61], and each country should tailor its policies to its own context.

Second, previous studies have shown that a delayed policy response will lead to higher mortality impact [14,15], whereas early and quick interventions with closure and containment policies are beneficial to controlling the COVID-19 pandemic and averting more deaths [16,17]. Our study goes beyond these findings and indicates that a quick first response always works well regardless of the country’s past epidemic experience and national income levels. For high-income countries, a quick response can concentrate economic resources on combating COVID-19, compensating for their lack of prior epidemic experience. For low-income countries, a speedy response can protect the elderly from risks of infection and death, compensating for their lack of economic resources.

Third, more emphasis should be placed on the elderly. It is well known that the elderly populations are more vulnerable to COVID-19 than younger people [64], and many studies have demonstrated the positive relationship between the proportion of elderly in the population and COVID-19 mortality impact [23,24]. Our research further shows that a large proportion of elderly in the population in conjunction with high population density may undermine the effectiveness of a country’s policy response and result in worse consequences. Therefore, more attention should be paid to protecting the elderly from COVID-19, especially in high-income countries with high population density or past epidemic experience.

### 4.3 Limitations

This study has three limitations. First, our sample did not cover all countries due to the availability of data, despite our efforts to construct a relatively large dataset including 80 countries, and this may constrain the generalizability of our findings. Second, through a process of model refinement, we only chose five factors of theoretical and empirical importance because the QCA method has a limitation on handling a large number of conditions under a limited number of cases. As a result, some macro or micro factors were excluded. Third, we used YLL rate rather than WLL rate as the outcome in light of the availability of data and being comparable to previous studies. When data is available, future studies should use WLL to perform a more fine-grained analysis of the mortality impact of COVID-19 [8,9].

## 5. Conclusion

This study offers a configurational lens to unpack the complexity involved in multiple interacting factors that together contribute to early COVID-19 mortality impact. An fsQCA investigation of 80 countries yielded eight combinations of five factors—a delayed public-health response, past epidemic experience, proportion of elderly in population, population density, and national income per capita—leading to either high or low early YLL rates. Some countries have fared better differently, whereas other countries have fared worse differently. Understanding how these factors configurate in various manners resulting in high or low YLL rates can help policy makers calibrate more contextualized and holistic strategies for combating future epidemics.

## Supporting information

S1 Table Dataset of 80 countries. (XLSX)

S2 Table Calibrated membership scores of 80 countries. (DOC)

S3 Table Necessity of conditions in explaining high and low YLL rates. (DOC)

S4 Table Truth table of high YLL rate. (DOC)

S5 Table Truth table of low YLL rate. (DOC)

S6 Table Multivariate regression results. (DOC)

S1 File Plots of models of the subsample using data from the holdout sample. (DOC)
