# Supplementary material for: Why were some countries more successful than others in curbing early COVID-19 mortality impact? A cross-country configurational analysis
Source: PLoS One. 2023 Mar 8;18(3):e0282617. doi: 10.1371/journal.pone.0282617 (PMC9994757; doi:10.1371/journal.pone.0282617)
Supplement: S2 Table — (DOC) [file pone.0282617.s002.doc]

**S2 Table. Calibrated membership scores of 80 countries.**

| ID | Country | A delayed public-health response | Past epidemic experience | Proportion of elderly in population | Population density | National income per capita | YLL rate |
| --- | --- | --- | --- | --- | --- | --- | --- |
| 1 | Afghanistan | 0.43 | 0 | 0.05 | 0.201 | 0.041 | 0.161 |
| 2 | Albania | 0.47 | 0 | 0.59 | 0.511 | 0.131 | 0.691 |
| 3 | Algeria | 0.88 | 1 | 0.13 | 0.061 | 0.101 | 0.081 |
| 4 | Argentina | 0.9 | 1 | 0.37 | 0.061 | 0.501 | 0.921 |
| 5 | Australia | 0.1 | 1 | 0.72 | 0.041 | 0.921 | 0.081 |
| 6 | Austria | 0.87 | 1 | 0.88 | 0.521 | 0.901 | 0.551 |
| 7 | Bangladesh | 0.05 | 1 | 0.09 | 1 | 0.071 | 0.291 |
| 8 | Belgium | 0.74 | 0 | 0.88 | 0.911 | 0.881 | 0.951 |
| 9 | Bolivia | 0.91 | 1 | 0.16 | 0.051 | 0.091 | 0.051 |
| 10 | Brazil | 0.67 | 1 | 0.25 | 0.081 | 0.311 | 0.971 |
| 11 | Burkina Faso | 0.91 | 1 | 0.04 | 0.301 | 0.051 | 0.051 |
| 12 | Cameroon | 0.93 | 1 | 0.05 | 0.181 | 0.061 | 0.041 |
| 13 | Canada | 0.05 | 1 | 0.82 | 0.041 | 0.871 | 0.551 |
| 14 | Chad | 0.85 | 0 | 0.05 | 0.051 | 0.051 | 0.051 |
| 15 | Chile | 0.94 | 1 | 0.41 | 0.081 | 0.531 | 0.941 |
| 16 | China | 0.01 | 1 | 0.38 | 0.601 | 0.331 | 0.041 |
| 17 | Colombia | 0.45 | 1 | 0.22 | 0.141 | 0.171 | 0.981 |
| 18 | Costa Rica | 0.88 | 1 | 0.28 | 0.501 | 0.481 | 0.751 |
| 19 | Croatia | 0.13 | 0 | 0.93 | 0.281 | 0.531 | 0.311 |
| 20 | Cuba | 0.39 | 1 | 0.7 | 0.521 | 0.281 | 0.051 |
| 21 | Cyprus | 0.88 | 0 | 0.58 | 0.561 | 0.711 | 0.121 |
| 22 | Czech Republic | 0.07 | 0 | 0.91 | 0.581 | 0.621 | 0.911 |
| 23 | Denmark | 0.71 | 0 | 0.91 | 0.591 | 0.951 | 0.531 |
| 24 | Dominican Republic | 0.95 | 1 | 0.16 | 0.731 | 0.241 | 0.331 |
| 25 | Ecuador | 0.67 | 1 | 0.16 | 0.271 | 0.171 | 0.931 |
| 26 | El Salvador | 0.1 | 1 | 0.21 | 0.851 | 0.101 | 0.061 |
| 27 | Estonia | 0.91 | 0 | 0.91 | 0.091 | 0.631 | 0.231 |
| 28 | Eswatini | 0.08 | 0 | 0.07 | 0.251 | 0.101 | 0.521 |
| 29 | Ethiopia | 0.08 | 0 | 0.06 | 0.501 | 0.051 | 0.041 |
| 30 | Finland | 0.15 | 0 | 0.96 | 0.061 | 0.891 | 0.321 |
| 31 | France | 0.05 | 1 | 0.92 | 0.551 | 0.841 | 0.801 |
| 32 | Germany | 0.49 | 1 | 0.95 | 0.761 | 0.891 | 0.651 |
| 33 | Greece | 0.6 | 1 | 0.95 | 0.371 | 0.601 | 0.671 |
| 34 | Haiti | 0.13 | 1 | 0.09 | 0.931 | 0.061 | 0.101 |
| 35 | Hungary | 0.87 | 0 | 0.9 | 0.521 | 0.541 | 0.131 |
| 36 | Iceland | 0.08 | 0 | 0.67 | 0.041 | 0.961 | 0.521 |
| 37 | India | 0.07 | 1 | 0.13 | 0.951 | 0.061 | 0.421 |
| 38 | Indonesia | 0.03 | 1 | 0.12 | 0.591 | 0.101 | 0.511 |
| 39 | Iraq | 0.37 | 0 | 0.06 | 0.431 | 0.131 | 0.051 |
| 40 | Ireland | 0.91 | 1 | 0.59 | 0.281 | 0.941 | 0.631 |
| 41 | Israel | 0.11 | 0 | 0.43 | 0.931 | 0.851 | 0.661 |
| 42 | Italy | 0.05 | 1 | 0.97 | 0.701 | 0.781 | 0.831 |
| 43 | Jamaica | 0.1 | 1 | 0.23 | 0.811 | 0.131 | 0.511 |
| 44 | Japan | 0.01 | 0 | 0.99 | 0.891 | 0.851 | 0.051 |
| 45 | Kenya | 0.04 | 0 | 0.04 | 0.441 | 0.061 | 0.051 |
| 46 | Latvia | 0.52 | 0 | 0.92 | 0.091 | 0.561 | 0.071 |
| 47 | Lithuania | 0.91 | 0 | 0.92 | 0.141 | 0.581 | 0.271 |
| 48 | Luxembourg | 0.87 | 0 | 0.6 | 0.781 | 0.981 | 0.541 |
| 49 | Malawi | 0.97 | 0 | 0.05 | 0.691 | 0.041 | 0.061 |
| 50 | Malta | 0.43 | 0 | 0.93 | 1 | 0.711 | 0.641 |
| 51 | Mexico | 0.56 | 1 | 0.16 | 0.241 | 0.311 | 0.991 |
| 52 | Moldova | 0.49 | 0 | 0.42 | 0.451 | 0.101 | 0.891 |
| 53 | Nepal | 0.06 | 0 | 0.11 | 0.701 | 0.051 | 0.071 |
| 54 | Netherlands | 0.88 | 1 | 0.9 | 0.971 | 0.911 | 0.771 |
| 55 | New Zealand | 0.11 | 1 | 0.73 | 0.061 | 0.851 | 0.051 |
| 56 | Nicaragua | 0.39 | 1 | 0.1 | 0.181 | 0.061 | 0.111 |
| 57 | Nigeria | 0.97 | 1 | 0.05 | 0.731 | 0.061 | 0.051 |
| 58 | Norway | 0.88 | 0 | 0.8 | 0.051 | 0.981 | 0.181 |
| 59 | Pakistan | 0.1 | 0 | 0.07 | 0.821 | 0.061 | 0.061 |
| 60 | Panama | 0.05 | 1 | 0.2 | 0.201 | 0.531 | 0.621 |
| 61 | Peru | 0.8 | 1 | 0.2 | 0.081 | 0.181 | 0.991 |
| 62 | Philippines | 0.1 | 1 | 0.1 | 0.901 | 0.101 | 0.501 |
| 63 | Poland | 0.87 | 0 | 0.85 | 0.551 | 0.531 | 0.091 |
| 64 | Portugal | 0.87 | 0 | 0.96 | 0.531 | 0.641 | 0.741 |
| 65 | Romania | 0.07 | 1 | 0.87 | 0.381 | 0.451 | 0.201 |
| 66 | Sierra Leone | 0.95 | 1 | 0.05 | 0.521 | 0.041 | 0.061 |
| 67 | Slovak Republic | 0.8 | 0 | 0.74 | 0.531 | 0.591 | 0.561 |
| 68 | Slovenia | 0.82 | 0 | 0.92 | 0.511 | 0.671 | 0.931 |
| 69 | South Africa | 0.05 | 1 | 0.1 | 0.151 | 0.181 | 0.151 |
| 70 | South Korea | 0.1 | 1 | 0.66 | 0.971 | 0.771 | 0.061 |
| 71 | Spain | 0.87 | 1 | 0.9 | 0.461 | 0.731 | 0.921 |
| 72 | Suriname | 0.93 | 1 | 0.15 | 0.041 | 0.161 | 0.471 |
| 73 | Sweden | 0.67 | 1 | 0.92 | 0.081 | 0.931 | 0.751 |
| 74 | Switzerland | 0.47 | 1 | 0.87 | 0.731 | 0.981 | 0.811 |
| 75 | Togo | 0.95 | 0 | 0.05 | 0.601 | 0.051 | 0.061 |
| 76 | Turkey | 0.06 | 1 | 0.22 | 0.521 | 0.391 | 0.511 |
| 77 | Ukraine | 0.91 | 0 | 0.77 | 0.321 | 0.081 | 0.741 |
| 78 | United Kingdom | 0.93 | 1 | 0.86 | 0.811 | 0.851 | 0.931 |
| 79 | United States | 0.11 | 1 | 0.74 | 0.111 | 0.951 | 0.941 |
| 80 | Uruguay | 0.93 | 0 | 0.65 | 0.061 | 0.571 | 0.081 |
