# Supplementary material for: Why were some countries more successful than others in curbing early COVID-19 mortality impact? A cross-country configurational analysis
Source: PLoS One. 2023 Mar 8;18(3):e0282617. doi: 10.1371/journal.pone.0282617 (PMC9994757; doi:10.1371/journal.pone.0282617)
Supplement: S3 Table — (DOC) [file pone.0282617.s003.doc]

**S3 Table. Necessity of conditions in explaining high and low YLL rates.**

| Condition | YLL rate | ~ YLL rate |
| --- | --- | --- |
|  | Consistency | Consistency |
| A delayed public-health response | 0.648 | 0.561 |
| Past epidemic experience | 0.649 | 0.518 |
| Proportion of elderly in population | 0.711 | 0.508 |
| Population density | 0.595 | 0.554 |
| National income per capita | 0.685 | 0.461 |
| ~ A delayed public-health response | 0.564 | 0.604 |
| ~ Past epidemic experience | 0.351 | 0.482 |
| ~ Proportion of elderly in population | 0.524 | 0.675 |
| ~ Population density | 0.688 | 0.667 |
| ~ National income per capita | 0.557 | 0.728 |

Note: ~ means the logical negation. For example: ~ A delayed public-health response = negation / absence of a delayed public-health response
