# Supplementary material for: Why were some countries more successful than others in curbing early COVID-19 mortality impact? A cross-country configurational analysis
Source: PLoS One. 2023 Mar 8;18(3):e0282617. doi: 10.1371/journal.pone.0282617 (PMC9994757; doi:10.1371/journal.pone.0282617)
Supplement: S4 Table — (DOC) [file pone.0282617.s004.doc]

**S4 Table. Truth table of high YLL rate.**

| No. | A delayed public-health response | Past epidemic experience | Proportion of elderly in population | Population density | National income per capita | Raw Consist. | PRI Consist. | Case |
| --- | --- | --- | --- | --- | --- | --- | --- | --- |
| 1 | 0 | 0 | 0 | 1 | 1 | 0.954 | 0.680 | Israel |
| 2 | 0 | 0 | 1 | 1 | 0 | 0.947 | 0.809 | Albania |
| 3 | 1 | 1 | 1 | 0 | 1 | 0.947 | 0.882 | Greece; Ireland; Spain; Sweden |
| 4 | 1 | 1 | 1 | 1 | 1 | 0.941 | 0.865 | Austria; Netherlands; United Kingdom |
| 5 | 1 | 1 | 0 | 0 | 1 | 0.928 | 0.841 | Argentina; Chile |
| 6 | 0 | 0 | 1 | 1 | 1 | 0.843 | 0.560 | Czech Republic; Japan; Malta |
| 7 | 0 | 1 | 0 | 0 | 1 | 0.825 | 0.564 | Panama |
| 8 | 0 | 1 | 1 | 1 | 1 | 0.822 | 0.598 | France; Germany; Italy; South Korea; Switzerland |
| 9 | 1 | 0 | 1 | 1 | 1 | 0.803 | 0.545 | Belgium; Cyprus; Denmark; Hungary; Luxembourg; Poland; Portugal; Slovak Republic; Slovenia |
| 10 | 0 | 1 | 1 | 0 | 0 | 0.779 | 0.430 | Romania |
| 11 | 0 | 1 | 1 | 1 | 0 | 0.776 | 0.323 | Cuba |
| 12 | 0 | 0 | 1 | 0 | 1 | 0.751 | 0.269 | Croatia; Finland; Iceland |
| 13 | 0 | 1 | 1 | 0 | 1 | 0.736 | 0.478 | Australia; Canada; New Zealand; United States |
| 14 | 0 | 1 | 0 | 0 | 0 | 0.735 | 0.468 | Colombia; Nicaragua; South Africa |
| 15 | 1 | 0 | 1 | 0 | 0 | 0.717 | 0.406 | Ukraine |
| 16 | 1 | 0 | 0 | 1 | 0 | 0.646 | 0.221 | Malawi; Togo |
| 17 | 1 | 1 | 0 | 1 | 0 | 0.632 | 0.307 | Costa Rica; Dominican Republic; Nigeria; Sierra Leone |
| 18 | 1 | 1 | 0 | 0 | 0 | 0.617 | 0.475 | Algeria; Bolivia; Brazil; Burkina Faso; Cameroon; Ecuador; Mexico; Peru; Suriname |
| 19 | 1 | 0 | 1 | 0 | 1 | 0.584 | 0.175 | Estonia; Latvia; Lithuania; Norway; Uruguay |
| 20 | 0 | 0 | 0 | 0 | 0 | 0.575 | 0.186 | Afghanistan; Eswatini; Iraq; Kenya; Moldova |
| 21 | 0 | 1 | 0 | 1 | 0 | 0.574 | 0.148 | Bangladesh; China; El Salvador; Haiti; India; Indonesia; Jamaica; Philippines; Turkey |
| 22 | 1 | 0 | 0 | 0 | 0 | 0.562 | 0.173 | Chad |
| 23 | 0 | 0 | 0 | 1 | 0 | 0.555 | 0.162 | Ethiopia; Nepal; Pakistan |
