# Supplementary material for: Why were some countries more successful than others in curbing early COVID-19 mortality impact? A cross-country configurational analysis
Source: PLoS One. 2023 Mar 8;18(3):e0282617. doi: 10.1371/journal.pone.0282617 (PMC9994757; doi:10.1371/journal.pone.0282617)
Supplement: S5 Table — (DOC) [file pone.0282617.s005.doc]

**S5 Table. Truth table of low YLL rate.**

| No. | A delayed public-health response | Past epidemic experience | Proportion of elderly in population | Population density | National income per capita | Raw Consist. | PRI Consist. | Case |
| --- | --- | --- | --- | --- | --- | --- | --- | --- |
| 1 | 0 | 0 | 0 | 1 | 0 | 0.914 | 0.838 | Ethiopia; Nepal; Pakistan |
| 2 | 1 | 0 | 1 | 0 | 1 | 0.912 | 0.825 | Estonia; Latvia; Lithuania; Norway; Uruguay |
| 3 | 1 | 0 | 0 | 0 | 0 | 0.909 | 0.827 | Chad |
| 4 | 0 | 0 | 0 | 1 | 1 | 0.902 | 0.320 | Israel |
| 5 | 1 | 0 | 0 | 1 | 0 | 0.899 | 0.779 | Malawi; Togo |
| 6 | 0 | 1 | 1 | 1 | 0 | 0.893 | 0.677 | Cuba |
| 7 | 0 | 0 | 0 | 0 | 0 | 0.866 | 0.743 | Afghanistan; Eswatini; Iraq; Kenya; Moldova |
| 8 | 0 | 0 | 1 | 0 | 1 | 0.852 | 0.567 | Croatia; Finland; Iceland |
| 9 | 0 | 1 | 1 | 0 | 0 | 0.833 | 0.570 | Romania |
| 10 | 1 | 1 | 0 | 1 | 0 | 0.828 | 0.675 | Costa Rica; Dominican Republic; Nigeria; Sierra Leone |
| 11 | 0 | 1 | 0 | 1 | 0 | 0.807 | 0.614 | Bangladesh; China; El Salvador; Haiti; India; Indonesia; Jamaica; Philippines; Turkey |
| 12 | 1 | 0 | 1 | 0 | 0 | 0.807 | 0.594 | Ukraine |
| 13 | 0 | 0 | 1 | 1 | 1 | 0.800 | 0.440 | Czech Republic; Japan; Malta |
| 14 | 0 | 0 | 1 | 1 | 0 | 0.776 | 0.191 | Albania |
| 15 | 0 | 1 | 0 | 0 | 1 | 0.774 | 0.436 | Panama |
| 16 | 0 | 1 | 0 | 0 | 0 | 0.768 | 0.532 | Colombia; Nicaragua; South Africa |
| 17 | 1 | 0 | 1 | 1 | 1 | 0.747 | 0.417 | Belgium; Cyprus; Denmark; Hungary; Luxembourg; Poland; Portugal; Slovak Republic; Slovenia |
| 18 | 0 | 1 | 1 | 1 | 1 | 0.735 | 0.402 | France; Germany; Italy; South Korea; Switzerland |
| 19 | 0 | 1 | 1 | 0 | 1 | 0.729 | 0.465 | Australia; Canada; New Zealand; United States |
| 20 | 1 | 1 | 0 | 0 | 0 | 0.628 | 0.490 | Algeria; Bolivia; Brazil; Burkina Faso; Cameroon; Ecuador; Mexico; Peru; Suriname |
| 21 | 1 | 1 | 0 | 0 | 1 | 0.618 | 0.159 | Argentina; Chile |
| 22 | 1 | 1 | 1 | 1 | 1 | 0.610 | 0.101 | Austria; Netherlands; United Kingdom |
| 23 | 1 | 1 | 1 | 0 | 1 | 0.600 | 0.118 | Greece; Ireland; Spain; Sweden |
