# Supplementary material for: Why were some countries more successful than others in curbing early COVID-19 mortality impact? A cross-country configurational analysis
Source: PLoS One. 2023 Mar 8;18(3):e0282617. doi: 10.1371/journal.pone.0282617 (PMC9994757; doi:10.1371/journal.pone.0282617)
Supplement: S6 Table — (DOC) [file pone.0282617.s006.doc]

**S6 Table. Multivariate regression results.**

| Variables | YLL rate | |
| --- | --- | --- |
|  | Standardized Coefficients | *P*-values |
| A delayed public-health response | 0.203 | 0.069 |
| Past epidemic experience | 0.269 | 0.017 |
| Proportion of elderly in population | 0.255 | 0.084 |
| Population density | -0.050 | 0.653 |
| National income per capita | -0.032 | 0.822 |
| *R^2^* | 0.14 | |
| *N* | 80 | |
