# Supplementary material for: Why were some countries more successful than others in curbing early COVID-19 mortality impact? A cross-country configurational analysis
Source: PLoS One. 2023 Mar 8;18(3):e0282617. doi: 10.1371/journal.pone.0282617 (PMC9994757; doi:10.1371/journal.pone.0282617)
Supplement: S1 File — (DOC) [file pone.0282617.s007.doc]

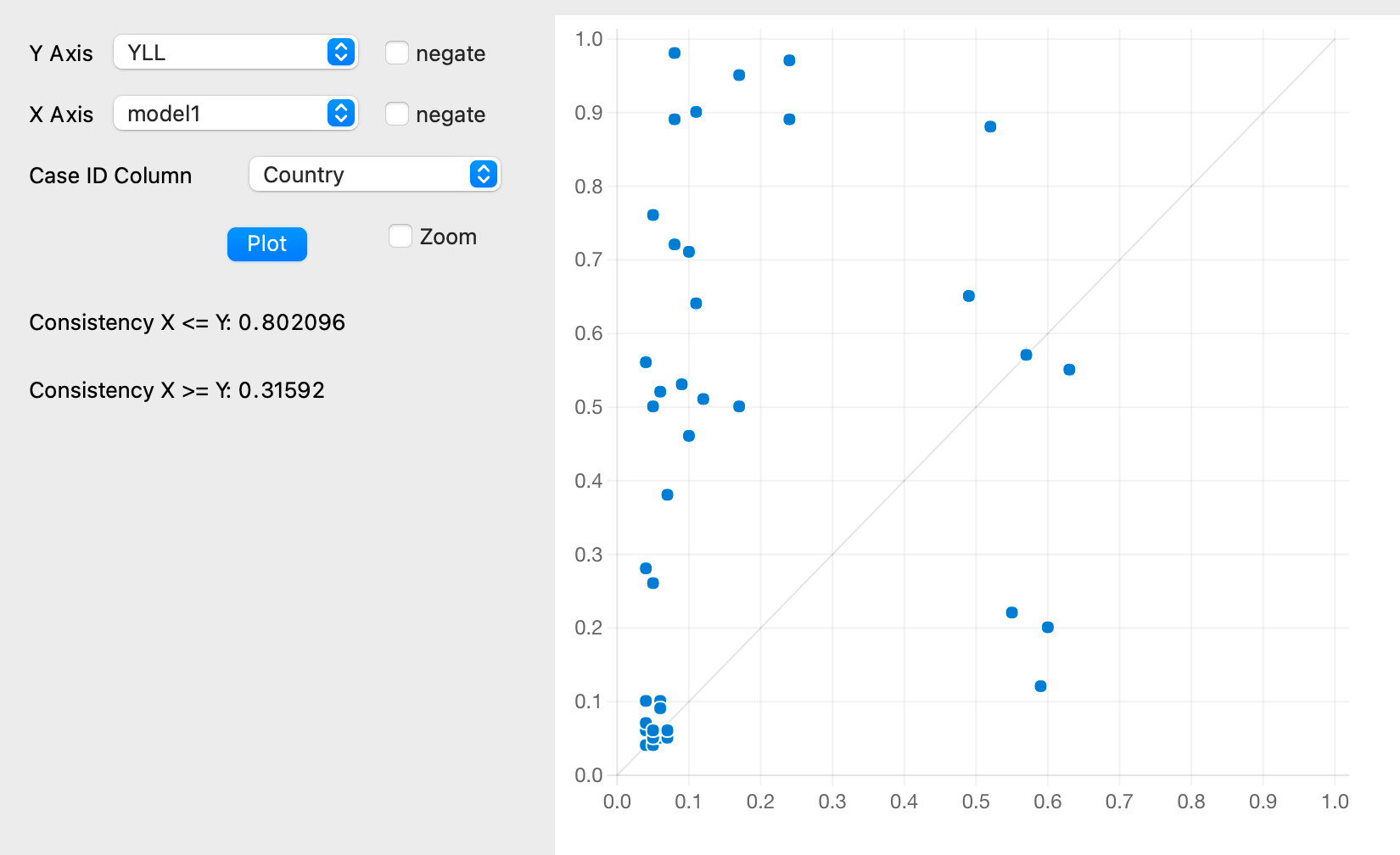


**S7a Fig. Plot of model 1 of the subsample using data from the holdout sample.**


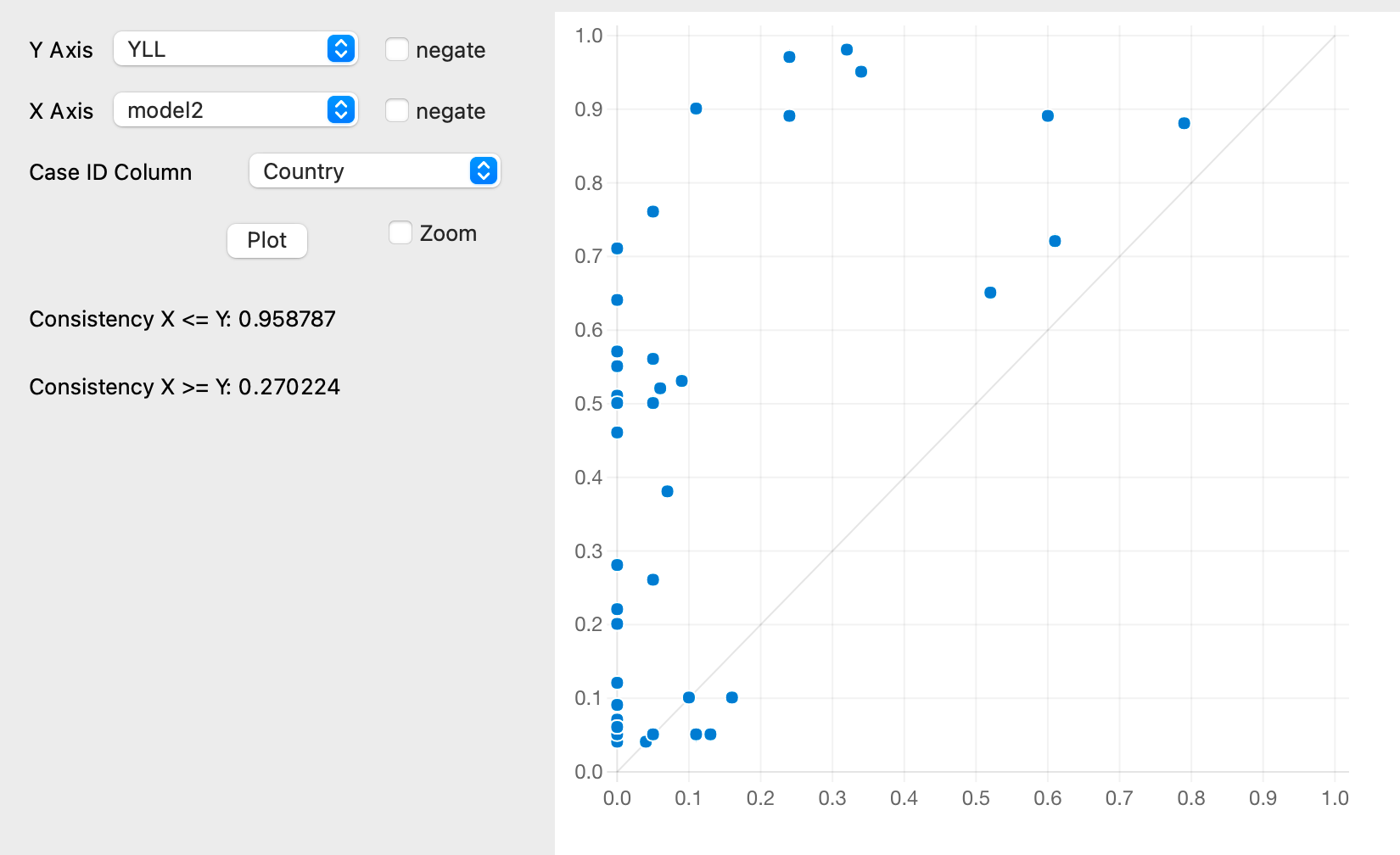


**S7b Fig. Plot of model 2 of the subsample using data from the holdout sample.**


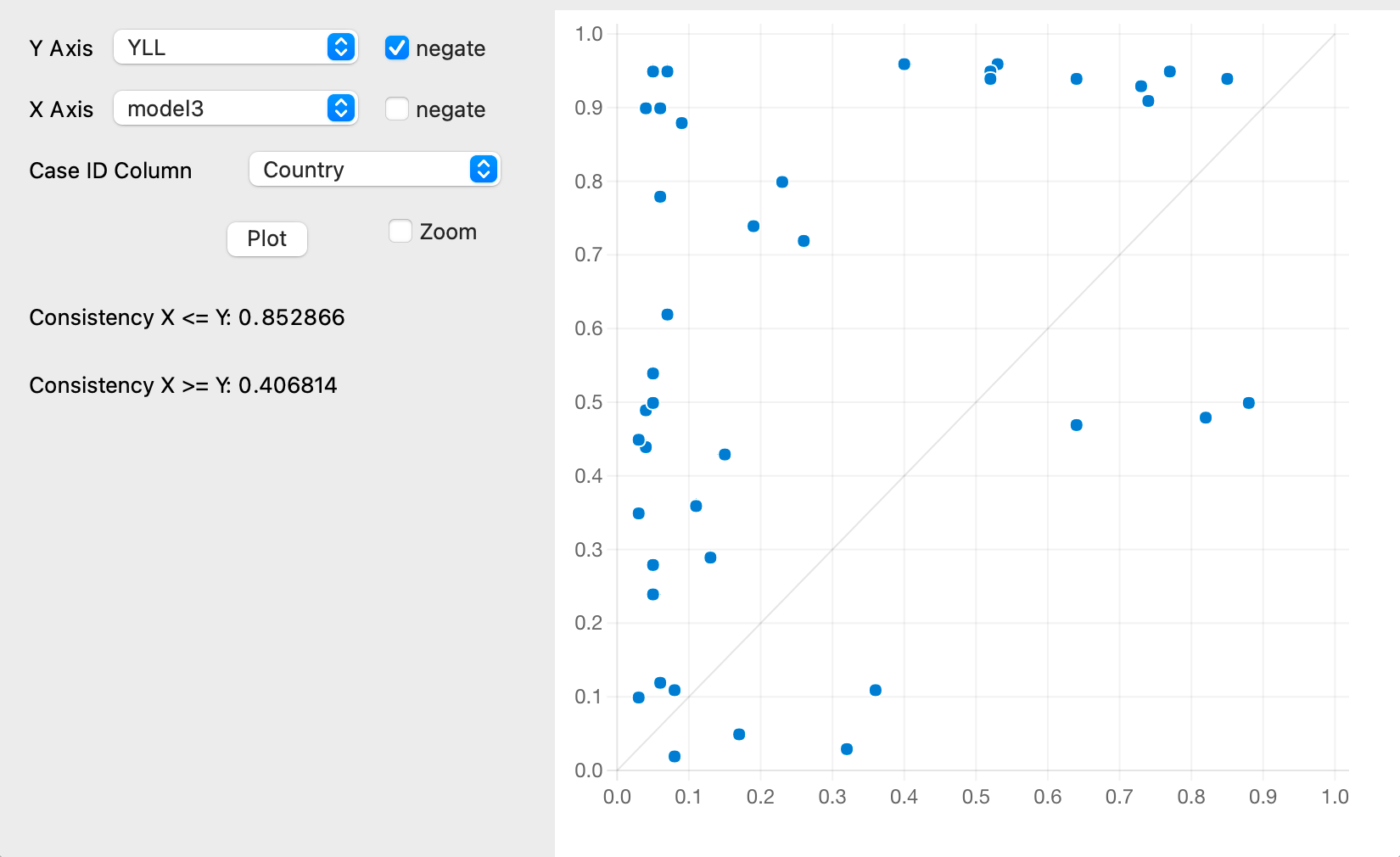


**S7c Fig. Plot of model 3 of the subsample using data from the holdout sample.**


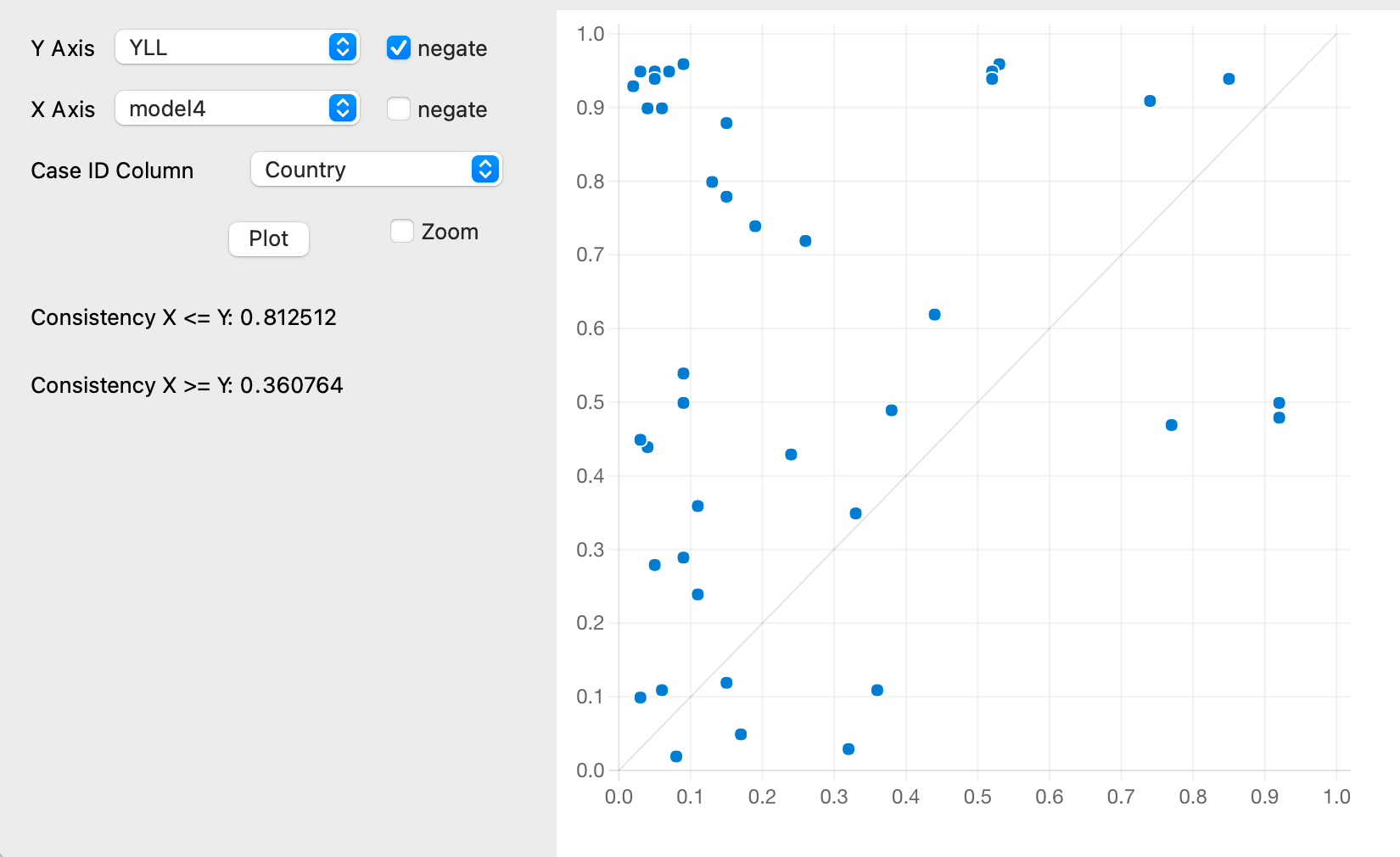


**S7d Fig. Plot of model 4 of the subsample using data from the holdout sample.**
